# Therapy-related myelodysplastic syndromes deserve specific diagnostic sub-classification and risk-stratification—an approach to classification of patients with t-MDS

**DOI:** 10.1038/s41375-020-0917-7

**Published:** 2020-06-29

**Authors:** A. Kuendgen, M. Nomdedeu, H. Tuechler, G. Garcia-Manero, R. S. Komrokji, M. A. Sekeres, M. G. Della Porta, M. Cazzola, A. E. DeZern, G. J. Roboz, D. P. Steensma, A. A. Van de Loosdrecht, R. F. Schlenk, J. Grau, X. Calvo, S. Blum, A. Pereira, P. Valent, D. Costa, A. Giagounidis, B. Xicoy, H. Döhner, U. Platzbecker, C. Pedro, M. Lübbert, I. Oiartzabal, M. Díez-Campelo, M. T. Cedena, S. Machherndl-Spandl, M. López-Pavía, C. D. Baldus, M. Martinez-de-Sola, R. Stauder, B. Merchan, A. List, C. Ganster, T. Schroeder, M. T. Voso, M. Pfeilstöcker, H. Sill, B. Hildebrandt, J. Esteve, B. Nomdedeu, F. Cobo, R. Haas, F. Sole, U. Germing, P. L. Greenberg, D. Haase, G. Sanz

**Affiliations:** 1 Department of Hematology, Oncology, and Clinical Immunology, University Hospital Duesseldorf, Duesseldorf, Germany; 2 Department of Laboratory Hematology, Institut Català d’Oncologia Hospital GermansTrias I Pujol, Badalona, Spain; 3 Boltzmann Institute for Leukemia Research, Hanusch Hospital, Vienna, Austria; 4 Department of Leukemia, MD Anderson Cancer Center, Houston, TX USA; 5 Department of Malignant Hematology, H Lee Moffitt Cancer Center, Tampa, FL USA; 6 Leukemia Program, Department of Hematology and Medical Oncology, Taussig Cancer Institute, Cleveland Clinic, Cleveland, OH USA; 7 Cancer Center - IRCCS Humanitas Research Hospital & Humanitas University, Rozzano - Milan, Italy; 8 Department of Hematology Oncology, IRCCS Policlinico San Matteo Foundation, Pavia, Italy; 9 Sidney Kimmel Comprehensive Cancer Center, Johns Hopkins University, Baltimore, MD USA; 10 Weill Cornell Medicine and The New York Presbyterian Hospital, New York, NY USA; 11 Dana-Farber Cancer Institute, Boston, MA USA; 12 Amsterdam UMC, Vrije Universiteit Amsterdam, Amsterdam, Netherlands; 13 Department of Internal Medicine III, University Hospital Ulm, Ulm, Germany; 14 National Center of Tumor Diseases-Trial Center, National Center of Tumor Diseases, German Cancer Research Center, Heidelberg, Germany; 15 Department of Internal Medicine V, Heidelberg University Hospital, Heidelberg, Germany; 16 Hematological Citology Laboratory, Pathology Department, Hospital del Mar, GRETNHE, IMIM Hospital del Mar Research Institute, Barcelona, Spain; 17 Service of Hematology, University Hospital Lausanne, Lausanne, Switzerland; 18 Hemotherapy and Hemostasis Department, Hospital Clínic de Barcelona IDIBAPS, Barcelona, Spain; 19 Department of Internal Medicine I, Division of Hematology & Hemostaseology and Ludwig Boltzmann Institute for Hematology and Oncology, Medical University of Vienna, Vienna, Austria; 20 Hematopathology Section, Hospital Clínic de Barcelona IDIBAPS, Barcelona, Spain; 21 Department of Oncology, Hematology and Palliative Care, Marienhospital Duesseldorf, Duesseldorf, Germany; 22 Clinical Hematology Department, Institut Català d’Oncologia, Hospital Germans Trias i Pujol, Badalona, Josep Carreras Leukemia Research Institute, Universitat Autònoma de Barcelona, Bellaterra, Spain; 23 University Hospital Leipzig, Leipzig, Germany; 24 Clinical Hematology Department, Hospital del Mar, Barcelona, Spain; 25 Department of Hematology, Oncology and Stem Cell Transplantation, University Medical Center Freiburg, Faculty of Medicine, Freiburg, Germany; 26 Clinical Hematology Department, Hospital Universitario Araba, Vitoria-Gasteiz, Spain; 27 Clinical Hematology Department, Hospital Universitario de Salamanca (HUSA), Salamanca, Spain; 28 Clinical Hematology Department, Hospital Universitario 12 de Octubre, Madrid, Spain; 29 1st. Internal Department – Hematology with stem cell transplants, Hemostaseology and Medical Oncology, Elisabethinen Hospital, Linz, Austria; 30 Clinical Hematology Department, Hospital General Universitari de València, Valencia, Spain; 31 Department of Hematology and Oncology, University Hospital Schleswig-Holstein, Campus Kiel, Kiel, Germany; 32 Clinical Hematology Department, Hospital Parc Taulí, Sabadell, Spain; 33 Department of Internal Medicine V (Hematology and Oncology), Innsbruck Medical University, Innsbruck, Austria; 34 Department of Hematology, University Hospital Vall d´Hebrón, Barcelona, Spain; 35 Department of Hematology and Medical Oncology, University Medical Center Göttingen, Göttingen, Germany; 36 Department of Biomedicine and Prevention, Tor Vergata University, Rome, Italy; 37 Hanusch Krankenhaus Wien, Vienna, Austria; 38 Medizinische Universität Graz, Graz, Austria; 39 Institute of Human Genetics, University Duesseldorf, Duesseldorf, Germany; 40 Clinical Hematology Department, Hospital Clínic de Barcelona, IDIBAPS, Barcelona, Spain; 41 Clinical Hematology Department, Hospital Quirón Teknon, Barcelona, Spain; 42 MDS Group, Josep Carreras Leukemia Research Institute, Barcelona, Spain; 43 Stanford University Cancer Center, Stanford, CA USA; 44 Clinical Hematology Department, Hospital Universitari I Politècnic la Fe, Valencia, Spain

**Keywords:** Risk factors, Diagnosis

## Abstract

In the current World Health Organization (WHO)-classification, therapy-related myelodysplastic syndromes (t-MDS) are categorized together with therapy-related acute myeloid leukemia (AML) and t-myelodysplastic/myeloproliferative neoplasms into one subgroup independent of morphologic or prognostic features. Analyzing data of 2087 t-MDS patients from different international MDS groups to evaluate classification and prognostication tools we found that applying the WHO classification for p-MDS successfully predicts time to transformation and survival (both *p* < 0.001). The results regarding carefully reviewed cytogenetic data, classifications, and prognostic scores confirmed that t-MDS are similarly heterogeneous as p-MDS and therefore deserve the same careful differentiation regarding risk. As reference, these results were compared with 4593 primary MDS (p-MDS) patients represented in the International Working Group for Prognosis in MDS database (IWG-PM). Although a less favorable clinical outcome occurred in each t-MDS subset compared with p-MDS subgroups, FAB and WHO-classification, IPSS-R, and WPSS-R separated t-MDS patients into differing risk groups effectively, indicating that all established risk factors for p-MDS maintained relevance in t-MDS, with cytogenetic features having enhanced predictive power. These data strongly argue to classify t-MDS as a separate entity distinct from other WHO-classified t-myeloid neoplasms, which would enhance treatment decisions and facilitate the inclusion of t-MDS patients into clinical studies.

## Introduction

Therapy-related myelodysplastic syndromes (t-MDS) are defined as MDS occurring as a complication of cytotoxic chemotherapy and/or radiation administered for an antecedent neoplastic or non-neoplastic disorder. According to the last World Health Organization (WHO)-classification, they belong to the group of therapy-related myeloid neoplasms (t-MNs) [1, 2]. From the first WHO-classification of myeloid disorders in 2001 [3], the WHO 2008 [4], to the current classification from 2016 [1, 2] patients with t-MDS have been considered as having a generally poor prognosis.

From the beginning, t-MDS patients were placed together into the large group of therapy-related myeloid neoplasms (t-MNs), independent of blast count and morphologic features such as cellularity or dysplasia. In the first WHO-classification, t-MNs were sub-classified based on causative agents: alkylating agent/ radiation-related versus topoisomerase-II inhibitor-related [3]. This subdivision was removed from the next WHO-classification [4], because in clinical practice it was difficult to apply, as many patients received combined chemotherapeutic regimens (alkylators, topoisomerase-II-inhibitors, antimetabolites, antitubulin agents) and/or radiation therapy.

The 2016 WHO-classification recognizes the fact that t-MNs can be sub-classified morphologically into t-MDS, t-MDS/MPN, and t-AML, but considers it best to distinguish them collectively from p-MNs as “a unique clinical syndrome” [1, 2], insinuating again that all t-MDS have a uniformly poor prognosis. If this were considered true, all treatable patients would need to receive a recommendation for an intensive/disease-modifying treatment approach, including allogeneic transplantation, chemotherapy, or hypomethylating agents.

Most prognostic tools for MDS have been developed excluding patients with t-MDS [5–7]. An exception is the MD Anderson Prognostic Scoring System (MDAPSS) [8]. Following the categorization of t-MDS as a non-separate subgroup of t-MN, most publications have analyzed a conglomerate of t-MDS and t-AML, usually including both treated and untreated patients [9–17]. Other publications [18–20] have focused on t-AML. In contrast, publications on t-MDS only are rare [21–23]. Not categorizing t-MDS as a subgroup of MDS limits proper clinical decision-making, interferes with epidemiological/ biological research, and supports the established practice of excluding t-MDS from clinical studies [24], thereby potentially preventing therapeutic improvements.

In cooperation with centers from the International Working Group for Prognosis in MDS (IWG-PM) as well as the U.S. MDS Clinical Research Consortium we have compiled a database comprising 2087 patients. After a detailed review of all ISCN formulas, strict criteria were applied regarding the cytogenetic data included in this analysis. Information existed on 1245 patients for overall survival (OS) and AML progression with complete reviewed data to apply IPSS-R and WHO-classification. For a comparison to primary MDS (p-MDS), we used data from 4593 patients from the IPSS-R database that was limited to the institutions contributing data to both projects.

These two very large databases on both t- and p-MDS enabled us to gather comparative prognostic data in t-MDS related to p-MDS, test the performance of currently existing tools for classification and prognostication, and finally improve the current stratification systems for use in t-MDS.

## Material and methods

Eight different study groups in the US, Germany, Spain, Italy, Austria, and the Netherlands contributed 2087 patients in total. By the contributing centers all patients with a diagnosis of MDS according to WHO and/or FAB were included if they had a history of an antecedent disease leading to chemo- (including alkylating agents, topoisomerase-II inhibitors, antimetabolites, and antitubulin agents) and/or radiotherapy [1–4, 25, 26]. To test the applicability and performance of different scoring systems as well as classifications strict inclusion/exclusion criteria were applied. Selection criteria included information about primary disease, pretreatment (at least chemotherapy or radiation), valid data to calculate the IPSS-R, survival data, AML transformation, valid stratification variables age, sex, and year of diagnosis. Patients were excluded if age <16 years, AML-defining cytogenetic abnormalities [inv(16), *t*(15;17), *t*(8;21)], peripheral blasts >19%, normal karyotypes based on <10 metaphases analyzed, proliferative CMML, AML as a primary diagnosis, survival or time to AML < 2 months occurred, and if the primary disease was in progression, to focus on the prognostic impact of the MDS itself. Karyotype was documented within the ISCN formula [27] after cytogenetic review (performed by DH, FS, JG, and BH). Treatment in MDS-phase, including intensive AML-type chemotherapy and allogeneic stem cell transplantation, was not an exclusion criterion, but the analyses were repeated with untreated patients only. These results are given in the supplement.

FAB, WHO-2016, IPSS-R, and WPSS-R classifications were calculated. For the WHO-classification RCUD, RARS, and MDS del(5q) were grouped together as in the WPSS. To test and quantify differentiating abilities of these tools the stratified log-rank test and the stratified Dxy-coefficient [28] were applied. Dxy is a concordance coefficient varying between −1 and 1, with 0 representing no monotone discriminative ability and 1 perfect monotone discrimination of a tool with respect to the time of interest (transformation free survival, overall survival, time to AML). As the main risk criterion, we used transformation-free survival since as a combined endpoint it described the clinically relevant disease-related risk more appropriately than overall survival. For completeness, Dxy values for overall survival and time to AML transformation were also included. In detail, time variables were defined as follows: all start with diagnosis of MDS and are censored, if no event occurred until the end of follow up. Transformation free survival ends when transformation or death without transformation occur. Time to AML ends with transformation but is censored in case of death without transformation. The results are based on stratified analyses compensating for possible confounding influences of sex, age, center, and year of diagnosis. Except for the influence of the primary diagnosis on the outcomes analyzed, we also stratified for primary diagnosis. Event time was calculated from time of MDS-diagnosis. Time to AML transformation was analyzed by censoring at time of death and in addition by treating death as competing event. Cumulative incidence curves for death with and without transformation are shown in the supplement.

All analyses were conducted with the statistics software R 3.4.3, including the package “survival” [29, 30]. Two-sided *P* values less than 0.05 were considered significant. In line with the essentially exploratory nature of the study, no adjustment for multiple testing was applied.

Data from t-MDS patients were compared with a cohort of 4593 untreated p-MDS patients from six different study groups within the IPSS-R database, including only patients from centers that also contributed cases to the t-MDS project.

## Results

### Patient characteristics

Of the total number of 2087 t-MDS patients, 1245 fulfilled all relevant selection criteria. Patients with high-risk (22%) and very high-risk (31%) IPSS-R score were more frequent among t-MDS than in the p-MDS group. Although less frequent, a considerable number (8% and 21% patients) had an IPSS-R of very low and low-risk, respectively (*p* < 0.001). Concordantly, as expected from previous publications [8], 30% t-MDS patients had a very poor and 15% a poor-risk cytogenetic score, and 27% had ≥5 abnormalities. Conversely, 2% and 37% patients were diagnosed with a very good or good-risk karyotype, respectively: 30% presented with a normal karyotype and 21% with only a single aberration. Patient characteristics as well as a comparison to p-MDS can be found in Table 1 and Supplementary Table 1 (the same analysis, limited to untreated patients is shown in Supplementary Table 2). Regarding WHO, the size of the four different risk groups was almost equal: RCUD, RARS, and MDS del(5q) 25%, RCMD 32%, RAEB-1 23%, and RAEB-2 21% and the distribution of the sub-groups were relatively similar to p-MDS (34%, 29%, 17%, and 20%). However, patients with RAEB-I were more frequent and RARS (6 vs. 13%), MDS del(5q) (1 vs. 4%), as well as MDS/MPN (CMML, excluding proliferative CMML) (4 vs. 10%) were less frequent (*p* < 0.001). The median overall survival was 18 months with a median follow up of 60 months for the t-MDS patients. The group of untreated p-MDS patients from the IPSS-R database had a median survival of 41 months. Median follow up was 49 months for this cohort, respectively.

**Table 1:** Patient characteristics of therapy-related (t-MDS) and primary (p-MDS) myelodysplastic syndromes (additional information on patient characteristics regarding participating centers and year of diagnosis and a comparison of untreated patients only is shown in Supplementary Tables 1 and 2).

Characteristics	t-MDS patients (*n* = 1245)		p-MDS patients (*n* = 4593)		*p*
	*n*	%	*n*	%	
MDS treatment					
Treated^a^	715	63%	0	0%	
Untreated	422	37%	4593	100%	
Total with information	1137	91%	4593	100%	
Stem cell transplantation					
Yes	210	19%	0	0%	
No	906	81%	4593	100%	
Total	1116	90%	4593		
Age (years)					<0.001
≤60	342	28%	1053	23%	
>60 to ≤70	395	32%	1267	27%	
>70 to ≤80	404	32%	1601	35%	
>80	104	8%	672	15%	
Median	68		70		
Total	1245	100%	4593	100%	
Gender					<0.001
Male	680	55%	2854	62%	
Female	565	45%	1739	38%	
Total	1245	100%	4593	100%	
FAB					<0.001
RA	490	40%	1707	37%	
RARS	110	9%	839	18%	
RAEB	455	38%	1217	27%	
RAEB-T	81	7%	328	7%	
CMML	44	4%	435	9%	
Unclassified	29	2%	67	2%	
Total	1209	97%	4593	100%	
WHO					<0.001
RCUD	183	17%	639	17%	
RARS	66	6%	507	13%	
RCMD	335	31%	1097	29%	
RAEB-1	246	22%	627	16%	
RAEB-2	219	20%	748	19%	
MDS (del5q)	13	1%	143	4%	
MDS-U	29	3%	90	2%	
Total	1091	88%	3851	84%	
IPSS-R					<0.001
Very low	105	8%	893	19%	
Low	260	21%	1644	36%	
Intermediate	225	18%	882	19%	
High	275	22%	628	14%	
Very high	380	31%	546	12%	
Total	1245	100%	4593	100%	
WPSS-R					<0.001
Very low	83	8%	822	22%	
Low	164	15%	1036	28%	
Intermediate	228	21%	654	17%	
High	399	38%	916	24%	
Very high	188	18%	325	9%	
Total	1062	85%	3753	82%	
Cytogenetic risk categories (IPSS-R)-cipssr					<0.001
Very good	24	2%	150	3%	
Good	460	37%	3261	71%	
Intermediate	198	16%	622	14%	
Poor	184	15%	197	4%	
Very poor	379	30%	363	8%	
Total	1245	100%	4593	100%	
Number of cytogenetic aberrations					<0.001
0	377	30%	2753	73%	
1	266	21%	706	19%	
2	144	12%	136	4%	
3	77	6%	55	2%	
4	50	4%	28	1%	
≥5	331	27%	76	2%	
Total	1245	100%	3754	82%	
Primary diagnosis					
Hematological	529	43%			
Breast	203	16%			
Prostate	125	10%			
Other solid tumor	342	28%			
Non-malignant disease	43	3%			
Total	1242	99,8%			
Years from primary diagnosis to MDS					
Median (years)	6.9				
≤2	130	11%			
>2 to ≤4	216	18%			
>4 to ≤8	347	29%			
>8 to ≤16	356	29%			
>16	166	14%			
Total	1215	98%			
Therapy for primary disease					
All chemotherapy	1000	80%			
All radiation including radioiodine	676	54%			
Radiation alone	243	19%			
Radioactive iodine	11	1%			
Chemotherapy alone	568	45%			
Radiation and chemotherapy	431	35%			
Total	1243	99,8%			
Alkylating agents					
Yes	536	65%			
No	292	35%			
Total	828	83%			
Topoisomerase II inhibitors					
Yes	356	43%			
No	472	57%			
Total	828	83%			
Antitubulin agents					
Yes	340	41%			
No	488	59%			
Total	828	83%			
Antimetabolites					
Yes	313	43%			
No	515	57%			
Total	828	83%			

^a^HMAs, chemotherapy, and/or allogeneic HSC transplant.

The t-MDS patients’ primary diagnoses were a solid tumor in 54% and a hematological disease in 43%. The remaining 13% of patients had received treatment for a benign immunological disease. Treatment included chemotherapy only in 46%, radiation only in 19%, and both in 35% of the patients.

### Application of classification and prognostic scoring systems

**Fig. 1:**
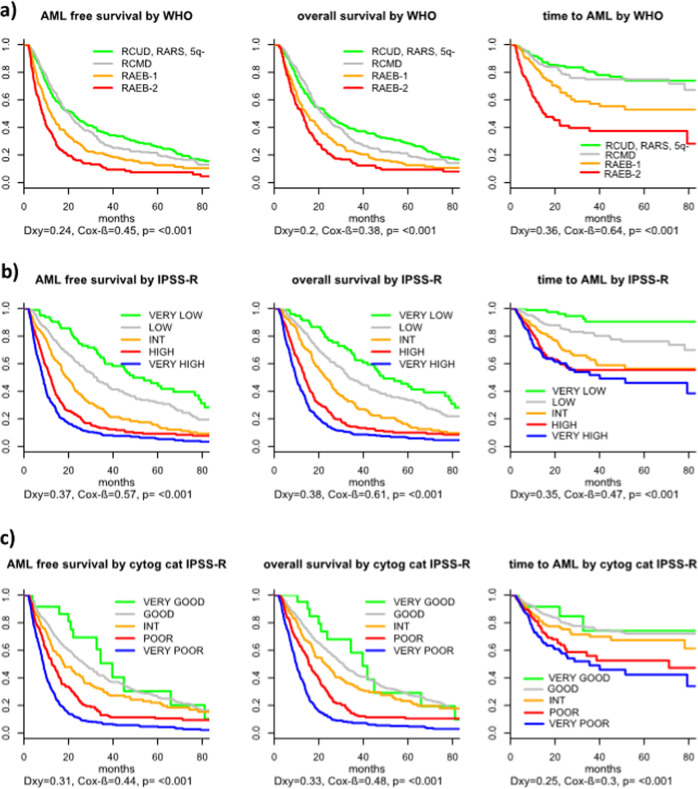
Outcome of patients with t-MDS according to different tools for classification and prognosis. **a** outcome according to WHO 2016, **b** outcome according to IPSS-R, **c** Outcome according to cytogenetic IPSS-R risk categories.

All classification and prognostic scoring systems applied were able to discriminate different risk groups within our t-MDS cohort, although the performance of the scoring systems was inferior when compared with p-MDS. The prognostic power measured by Dxy for the different classification and scoring systems in t- and p-MDS is given in Table 2 (the same analysis, limited to untreated patients only, is shown in Supplementary Table 3). The FAB-classification (Supplementary Fig. 1a) could only discriminate two different adjacent risk groups (RA vs. RAEB *p* < 0.001). There was no significant prognostic difference between RA and RARS (*p* = 0.8) as well as RAEB and RAEB-T (*p* = 0.291). However, these results are in line with results obtained in primary MDS and are thus not t-MDS specific. To examine the WHO-classification, we decided to use the categorization used within the WPSS, as there was no expectation regarding risk differences between RCUD, RARS, and MDS (del5q). With this combined low-risk group a better separation versus the remaining categories could be achieved, although there was no statistical significant difference between MDS with unilineage dysplasia (RCUD plus RARS plus MDS(del5q)) and MDS with multilineage dysplasia (*p* = 0.389), while RCMD vs. RAEB I (*p* < 0.001) and RAEB I vs RAEB II (*p* < 0.001) differed significantly (Fig. 1a). The same observation could be made in p-MDS (RCUD, RARS + MDS(del5q) vs. RCMD *p* = 0.403; RCMD vs. RAEB I *p* < 0.001; RAEB I vs RAEB II *p* < 0.001).

**Table 2:** Dxys for the different scoring systems and outcomes presented for t- and p-MDS (therapy-related and primary myelodysplastic syndromes): FAB (French-American-British classification), WHO (World Health Organization classification), IPSS-R (International Prognostic Scoring System-revised), WPSS-R (WHO-based Prognostic Scoring System-revised, cipssr (cytogenetic component of the IPSS-R), number of aberrations, and primary diagnosis.

Score	Transformation free survival		Overall survival		Time to AML	
	t-MDS	p-MDS	t-MDS	p-MDS	t-MDS	p-MDS
FAB	0.19	0.30	0.17	0.28	0.24	0.42
WHO	0.24	0.29	0.19	0.26	0.41	0.44
IPSS-R	0.37	0.41	0.38	0.40	0.36	0.53
WPSS-R	0.35	0.38	0.33	0.36	0.40	0.51
cipssr	0.30	0.23	0.32	0.23	0.23	0.28
Number of aberrations	0.29	0.13	0.32	0.13	0.22	0.14
Primary diagnosis	0.05	/	0.05	/	0.03	/

The two prognostic scoring systems performed both very well. The IPSS-R could separate five different risk groups for all outcomes tested (Fig. 1b), while the WPSS separated five risk groups regarding OS, but only four regarding PFS and AML transformation (see Supplementary Fig. 1b). The influence of IPSSR(A) is shown in Supplementary Fig. 1c. Regarding these outcomes, the difference did not reach statistical significance between low and very low-risk, likely because the very low-risk group was relatively small (*p* = 0.146).

When we analyzed the performance of the cytogenetic component of the IPSS-R (cipssr), the prognostic power was already very high on its own. Only the difference between the very low and low-risk group did not reach statistical significance (*p* = 0.210), but this might very likely be a matter of the size of the very low-risk group (*n* = 24). The performance of the cipssr was at least equal in t-MDS compared with p-MDS (Fig. 1c).

Other possible, t-MDS specific influences like the primary diagnosis (Dxy 0.05) or type of prior treatment did not influence the different outcomes significantly. Only patients with other, non-malignant disease appeared to have a better outcome (Supplementary Fig. 1d, *p* = 0.051). Cumulative incidence of death with and without transformation as an addition to Fig. 1a–c are shown in Supplementary Fig. 2a–c.

**Fig. 2:**
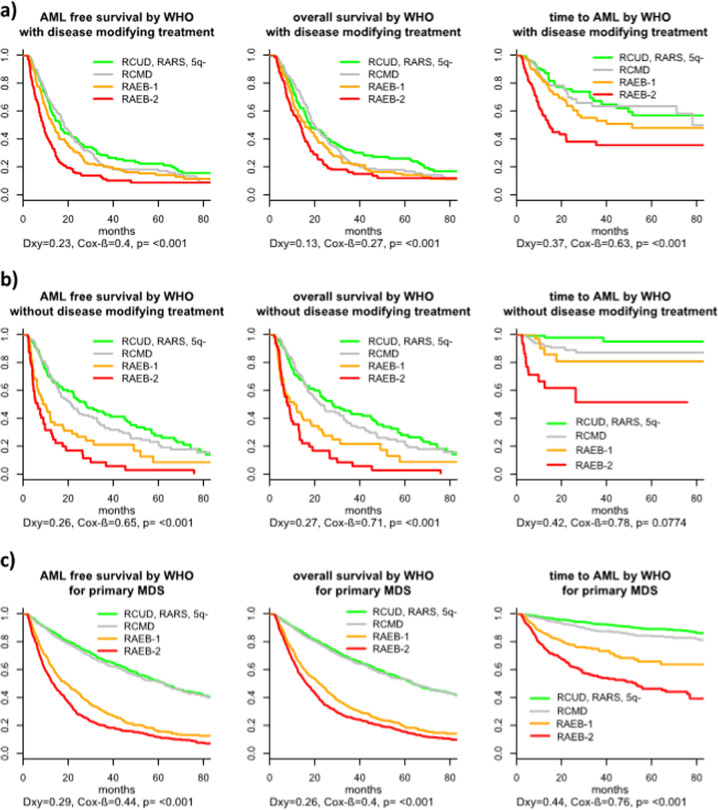
Outcome of patients with t-MDS according WHO-classification depending on treatment in MDS phase and comparison to p-MDS. **a** Outcome according to WHO-classification for treated patients. **b** Outcome according to WHO-classification for untreated patients. **c** Outcome according to WHO: p-MDS.

Since the prognostic values of all classification and scoring systems as well as single variables, except the cipssr, were inferior compared with p-MDS (Dxy (IPSS-R) in t-MDS 0.38 for OS and 0.35 for time to AML), we analyzed if this was influenced by the fact that our cohort was a mixture of treated and untreated patients. This hypothesis could be verified since all scoring systems performed much better in the subsample of untreated t-MDS patients (see Fig. 2a–c and Supplementary Fig. 3a–c). In this subgroup the Dxy for the IPSS-R was not inferior (0.45 for OS and 0.48 for time to AML) when compared with the p-MDS cohort (0.40 for OS and 0.53 for time to AML) and for the cipssr 0.30 and 0.54 in t-MDS versus 0.23 and 0.28 in p-MDS (Supplementary Table 3).

**Fig. 3:**
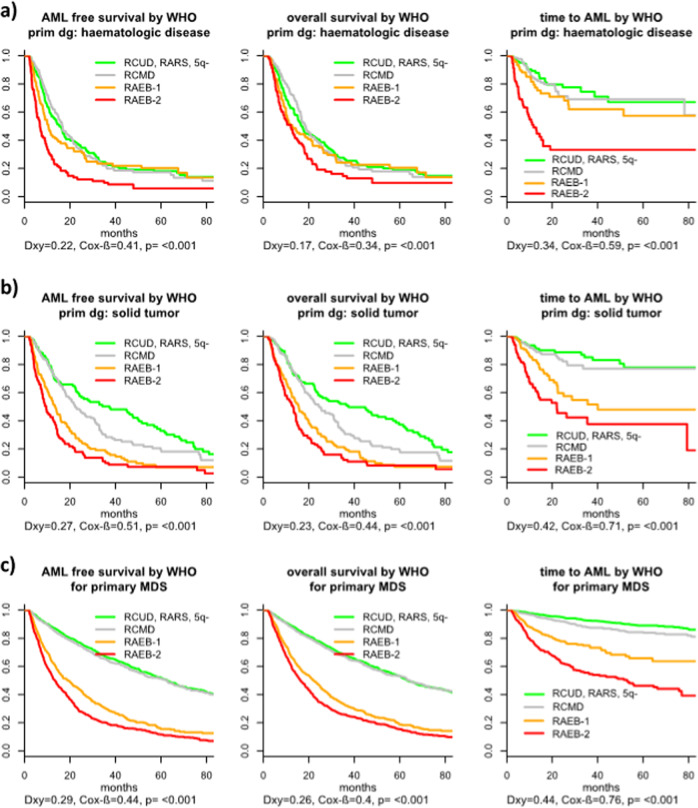
Outcome of patients with t-MDS according WHO-classification depending on the primary disease and comparison to p-MDS. **a** Outcome according to WHO: primary disease hematologic. **b** Outcome according to WHO: primary disease solid tumor **c** Outcome according to WHO**:** p-MDS.

Although patients with t-MDS in general could be classified by the WHO-classification system developed for p-MDS, its performance differed within specific t-MDS subgroups. The prognostic power was almost comparable to p-MDS in patients with a solid tumor as primary disease as well as in patients after radiotherapy only. In patients with a history of a hematologic disease or after chemotherapy, the prognostic power was lower. However, even in these subgroups, patients with different outcomes could be separated (see Fig. 3a–c and Supplementary Fig. 4a–c).

## Discussion

In this collaborative IWG-PM project, we were able to assemble the largest database on t-MDS to date, offering the analysis of well-characterized and clinically annotated data with a long follow up. Our analyses of the data presented here showed that patients with t-MDS benefited in a major way from receiving differentiated classification and prognostic evaluation, distinct from t-MN. Our scrupulous evaluation of the patients’ earlier treatment for malignant or non-malignant disease contributed to our case-finding.

T-MDS has been relatively neglected regarding classification and differentiated prognostication. All versions of the WHO-classification, including the latest from 2016, do not classify t-MDS within the group of MDS [1, 2]. Instead, t-MDS patients are currently still placed together with t-AML and t-MPD in a combined category of therapy-related myeloid neoplasms [1, 2]. Moreover, most established scoring systems were developed excluding t-MDS patients. The only t-MDS specific score was published by Quintas-Cardama et al. including variables such as performance status and age, which determine patient-related but not disease-specific risk features, and performance status might not be readily available in all databases [23].

Until now, only one paper analyzed the impact of the WHO-classification for p-MDS in t-MDS [21]. This publication by Singh et al. [21] included 155 patients with t-MDS or t-AML of whom only 81 patients were t-MDS. No differences were found in median survival times among patients classified into the different WHO subgroups and Singh and coworkers described a uniformly poor outcome in t-MDS regardless of morphologic classification. These results might be explained partially by the difference in size compared with our patient cohort. Furthermore, the paper includes patients not in remission from their primary disease. We excluded such patients to obtain a cleaner estimation of the specific MDS-related risk.

Earlier publications on t-MDS demonstrated mostly high-risk karyotypes in these patients, mainly including chromosome 5 and 7 abnormalities as well as complex karyotypes, in more than 90% of patients [9, 31, 32]. Although these features are still a hallmark of t-MDS in general, they do not represent all patients with a history of chemo- and/or radiotherapy. Our data demonstrated an unexpectedly high percentage of good-risk and normal cytogenetics, which are concordant with other more recently published data [13, 19, 23]. Regarding these early t-MDS publications, a reporting bias may have contributed since in some cases conspicuous cytogenetics might have been required for the question about previous treatments.

Even in the most recent WHO-classification [1, 2] the prognosis of t-MN is described as being generally poor and that prognosis of these patients is influenced strongly by karyotype as well as by the primary disease. Although this important information is given in the Revised Fourth Edition of the WHO series on histological and genetic typing of human tumors it remains purely descriptive, as it provides no consequences for the resulting t-MN classification. Chromosome 5 and/or 7 abnormalities, *TP53* mutations, and complex karyotype are indicated by the WHO as having a particularly poor outcome, while patients with balanced translocations were stated to have a good prognosis, albeit not as good as in p-MN. However, exceptions to these generalizations are notable. In our database we observed some patients with isolated del(5q) and a history of exposure to mutagenic agents that have a good prognosis, as is the case in p-MDS [33]. Conversely, considered typical for t-MDS is the group with balanced translocations involving chromosome band 11q23, the localization of the MLL/KMT2A-gene [9, 33–37]. These translocations can, in contrast to the typical good risk “AML defining translocations” t(8;21), t(15;17), and inv(16), occur as t-MDS and are associated with an extremely poor prognosis as previously described [9, 33–37]. These categorization problems relate in part from combining t-MDS and t-AML leading to the use of AML cytogenetic classification systems for the entire group of t-MN. In our study we have demonstrated that the influence of cytogenetics is very high in t-MDS and the prognostic power of the cipssr is in t-MDS at least as good as in p-MDS, due to a high proportion of abnormal karyotypes. Without a proper morphological classification and separation from t-AML, t-MDS patients are also withheld the most powerful tool for prognostic evaluation.

In addition, prognostic factors for t-MDS, other than cytogenetics, are not recognized by the WHO-classification. In practice, this means that a patient with a blast count of 0% (t-MDS) is in the same risk category as a patient with a blast count of 99% (t-AML). Also, with the current WHO-classification irrelevant for therapeutic decision-making is whether a patient has dysplastic (t-MDS) or proliferative disease (t-MDS/MPD). These issues have major implications for patient therapy. Thus, proper morphologic and cytogenetic classification as well as separation from t-AML, provide t-MDS patients a most powerful method for treatment considerations.

Our data demonstrated that t-MDS patients can be subdivided by diagnostic procedures into groups with clearly varying prognoses. These findings are underlined by results from smaller group analyses focusing on the value of prognostic scoring systems [22, 23, 38]. It should be considered in particular that sporadic MDS cases might be assigned to the group of t-MN based on their therapeutic history although this could be only coincidental, and they actually belong to p-MDS. Observational data on MDS patients cannot prove a causal link between therapy and a later developing MDS. This fact is already implied by the term “therapy-related”. As we cannot differentiate between the two situations this should not be taken as an argument for a less scrupulous diagnostic classification of the concerned patients. Quite the contrary, such spontaneously developed MDS within the t-MDS group have the greatest disadvantage from not being classified as p-MDS.

**Fig. 4:**
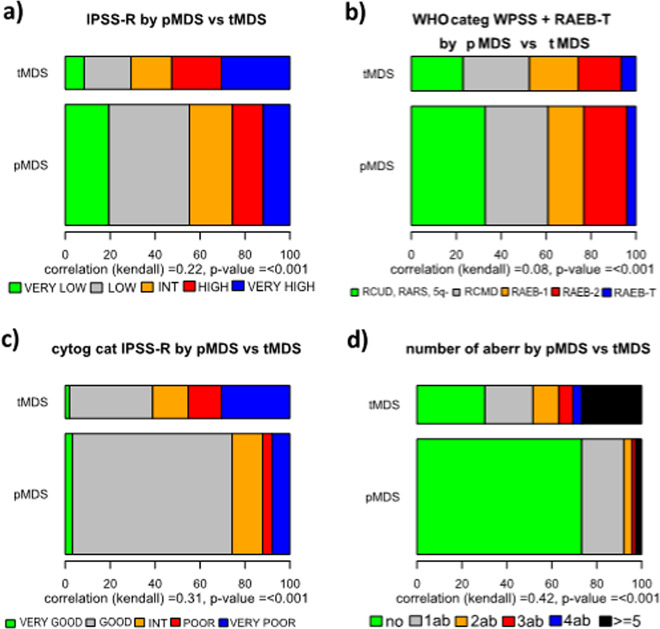
Distribution of risk groups according to different classification and prognostic tools for p- versus t-MDS. **a** Distribution of IPSS-R subgroups in p- and t-MDS. **b** Distribution of WHO- subtypes (according to WPSS + RAEB-T) in p- and t-MDS. **c** Distribution of cytogenetic IPSS-R subgroups in p- and t-MDS. **d** Distribution of number of aberrations in p- and t-MDS.

**Fig. 5:**
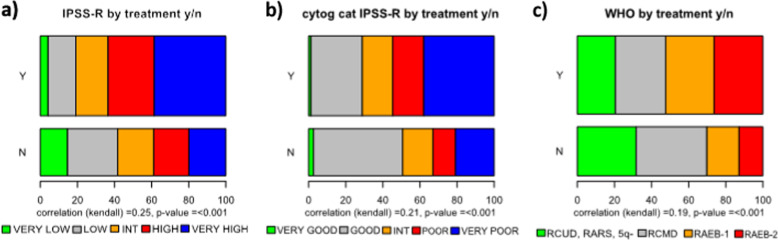
Distribution of risk groups according to different classification and prognostic tools for treated versus untreated patients. **a** Comparison of IPSS-R risk-groups between treated and untreated patients. **b** Comparison of IPSS-R cytogenetic risk-groups between treated and untreated patients. **c** Comparison of WHO subgroups between treated and untreated patients.

In addition, we note that the survival of t-MDS patients in different WHO groups was inferior to p-MDS, similar to findings in t- vs p-AML [18, 19]. This might have several reasons, including patient-related factors associated with the primary disease (relapse/progression of the primary disease, cumulative toxicity of primary and secondary therapy on bone marrow reserve as well as other organ function), as previously discussed [10, 19, 22, 39, 40]. A second point for discussion might be the retrospective nature of our, as well as previous studies. And third, in the present database, we can only compare data from a t-MDS group that is heterogeneous regarding treatment to an untreated group of p-MDS patients. As we have demonstrated, this influences the power of prognostic models and likewise will influence patient outcomes in general as well, although further analyses are needed to understand the exact nature of such effects. We find a relevant shift towards better risk patients in our t-MDS cohort if we look at untreated patients only. It is likely, that there will be a similar shift in the p-MDS cohort as well, since treated p-MDS patients were not included in our reference database (compare Figs. 4 and 5). Results in t- and p-MDS would possibly be better comparable if the analysis were restricted to untreated patients only. However, regarding the main issue of differentiating and sub-classifying tMDS exclusion of treated cases would bias those conclusions, since we observed a relevant shift towards older age and lower-risk disease among untreated patients (Fig. 5, Supplementary Table 2).

**Fig. 6:**
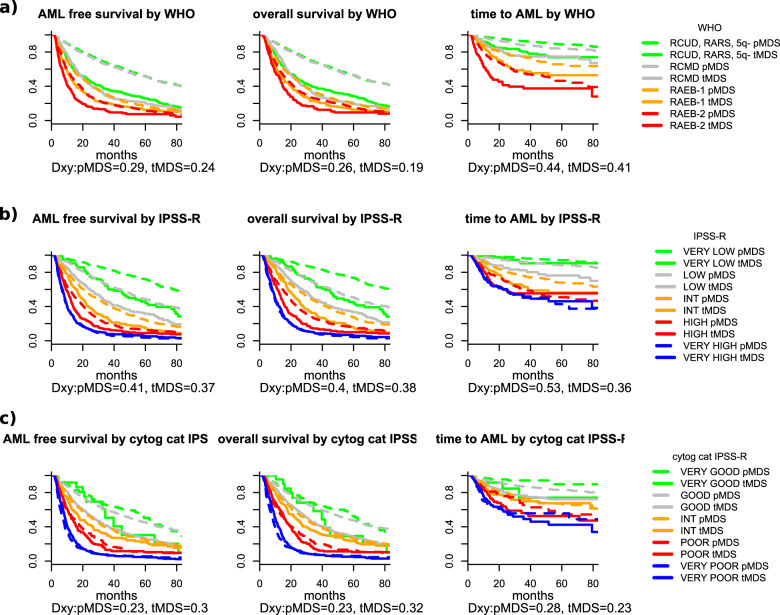
Comparison of outcome according to different tools for classification or prognostic evaluation t- versus p-MDS. **a** Comparison p- and t-MDS according to WHO-classification. **b** Comparison t- and p-MDS according to IPSS-R risk-categories. **c** Comparison t- and p-MDS according to cytogenetic IPSS-R categories.

Further, in addition to patient and method-related factors, biological differences between the leukemic stem cells might contribute as well, since inferior outcome of patients with t-MDS seems not only limited to survival. Especially in the good and intermediate-risk group, we observe this phenomenon with regard to AML transformation (see Fig. 6a–c and Supplementary Fig 5a-c). It is possible that, even within each subgroup there is a shift to higher risk cytogenetic or molecular abnormalities. This will be an important comparison for future analyzes. The impact of cytogenetics appears to be even greater in t-MDS compared with p-MDS. The major reason might be that the proportion of patients with aberrant karyotype was higher in t-MDS. In p-MDS, about half of the patients presented with a normal karyotype and 71% belonged to the large cipssr good risk group, whereas in t-MDS it was only 30 and 37%, respectively (see Table 1).

Regarding molecular differences, Singhal et al. found that t-MDS patients with ≥15% ringed sideroblasts had a low frequency of SF3B1 mutations, but a much higher frequency of TP53 mutations compared with patients with p-MDS [41]. In line with this observation the same analysis showed a generally much higher frequency of TP53 mutations in patients with t-MDS and about half of the patients had <5% marrow blasts at the time of t-MDS diagnosis [41]. Clonal hematopoiesis or germline predisposition can be found in hematopoietic cells of patients who develop t-MN, even before treatment of the prior disease, representing a sign of increased chromosomal instability and high-risk disease [16, 41–44]. However, preceding clonal hematopoiesis or genetic predisposition can also be found before the development of p-MDS [45–49].

Pedersen-Bjergaard et al. [50] suggested 25 years ago different genetic pathways for t-MDS and t-AML due to their differing distribution of genetic abnormalities. While this is the case for some t-MNs, in other cases, as has been evident with more recently developed mutational data, a biological continuum exists from clonal hematopoiesis to t-MDS and “secondary” t-AML with or without a MDS pre-phase and MDS-related features [16, 20, 41, 42]. These different mechanisms of leukemogenesis occur in therapy-related as in primary MNs.

Our data showed that, transformation-free survival (TFS) was poorer in t-MDS subgroups vs those in p-MDS: range 8–22 months, compared with 13–63 months, respectively. The impact of blast count and the performance of morphological classifications in t-MDS was somewhat less than in p-MDS, but remained substantial, in contrast to earlier, smaller publications [21]. Thus, all patients can and should be subdivided into different diagnostic subcategories and risk groups.

Knowledge of the diagnostic and prognostic evaluation of t-MN patients according to pathogenesis and disease characteristics is required for selecting patients who can be cured or will at least benefit from active treatment [10, 24] and would facilitate clinical as well as epidemiological research. An updated classification should separate t-MDS from t-AML and t-MDS/MPN as although these diseases might share some overlapping features, they exhibit differences in clinical presentation and molecular and cytogenetic characteristics [51, 52]. As for t-MDS, the feasibility of further sub-classification has been demonstrated by publications on t-AML as well [9, 18, 19, 37, 53–56]. Regarding t-MDS/MPN, only 4% of the patients in our database presented with t-CMML dysplastic, as its frequency is lower in t- compared with p-MDS [57]. Patients with t-CMML proliferative were excluded from this analysis. Our data indicate for all t-MDS an increased influence of karyotype on prognosis compared with other prognostic variables. This finding is similar to that demonstrated for p-CMML and t-CMML wherein the frequency of cytogenetic abnormalities is much higher in t-CMML, while the frequency of most molecular abnormalities seems to be comparable [57]. Based on this important publication, Patnaik et al. suggest that, due to the unique biological pattern and dismal prognostic impact, t-CMML should be considered as separate subtype in the classification scheme for both CMML and t-MN [57].

Based on our findings, we believe this newly established disease category t-MDS deserves further p-MDS-like subcategorization. We propose an approach using the WHO classification for p-MDS, but preceding each subgroup with a t-; for example, t-MDS-SLD, t-MDS-MLD, etc. We suggest restricting the use of prognostic systems to t-MDS patients in remission from their primary disease so as not to confound the results of risk factors for the MDS. For those not in remission it would be important to incorporate the confounding risk of the primary disease into clinical decision-making.

In summary, our data demonstrated that classification tools established in p-MDS were effective for stratifying subgroups in t-MDS and indicated the high prognostic relevance of cytogenetics in t-MDS. These findings from the largest t-MDS database to date should initiate a discussion of a potential revision of the WHO-classification and encourage clinicians to use the existing tools for risk assessment and treatment decisions for patients with therapy-related disease.

## Supplementary information

Supplementary Table 1

Supplementary Table 2

Supplementary Table 3

Supplementary Figure Legends

Supplementary Figure 1

Supplementary Figure 2

Supplementary Figure 3

Supplementary Figure 4

Supplementary Figure 5
