# Supplementary material for: Therapy-related myelodysplastic syndromes deserve specific diagnostic sub-classification and risk-stratification—an approach to classification of patients with t-MDS
Source: Leukemia. 2020 Jun 29;35(3):835–49. doi: 10.1038/s41375-020-0917-7 (PMC7932916; doi:10.1038/s41375-020-0917-7)
Supplement: Supplementary file 1 — Supplementary Table 1 [file 41375_2020_917_MOESM1_ESM.docx]

| **Characteristics** | **t-MDS patients (n=1245)** | | **p-MDS patients (n=4593)** | |
| --- | --- | --- | --- | --- |
|  | **n** | **%** | **n** | **%** |
| **Center (group)** |  |  |  |  |
| Austrian/German | 146 | 12% | 288 | 6% |
| Spain | 118 | 10% | 2227 | 49% |
| American research consortium | 351 | 28% | 173  (Cleveland only) | 4% |
| Graz | 14 | 1% | 0 | 0% |
| MD Anderson Texas | 565 | 45% | 1475 | 32% |
| Netherlands | 7 | 1% | 63 | 1% |
| Pavia | 44 | 4% | 367 | 8% |
| total | 1245 | 100% | 4593 | 100% |
|  |  |  |  |  |
| **Year of diagnosis** |  |  |  |  |
| >1960 to ≤1985 | 2 | 0,2% | 107 | 2% |
| >1985 to ≤1990 | 1 | 0,1% | 359 | 8% |
| >1990 to ≤1995 | 11 | 0,9% | 502 | 11% |
| >1995 to ≤2000 | 80 | 6,4% | 909 | 20% |
| >2000 to ≤2005 | 268 | 21,5% | 1489 | 32% |
| >2005 to ≤2010 | 494 | 39,7% | 1227 | 27% |
| >2010 to <2015 | 389 | 31,2% | 0 | 0% |
| total | 1245 | 100% | 4593 | 100% |
|  |  |  |  |  |

**Supplementary Table 1: Additional information on patient characteristics** regarding participating centers and year of diagnosis
