# Supplementary material for: Therapy-related myelodysplastic syndromes deserve specific diagnostic sub-classification and risk-stratification—an approach to classification of patients with t-MDS
Source: Leukemia. 2020 Jun 29;35(3):835–49. doi: 10.1038/s41375-020-0917-7 (PMC7932916; doi:10.1038/s41375-020-0917-7)
Supplement: Supplementary file 2 — Supplementary Table 2 [file 41375_2020_917_MOESM2_ESM.docx]

| **Characteristics** | **t-MDS patients (n=530)**  **untreated only** | | **p-MDS patients (n=4593)** | | ***p*** |
| --- | --- | --- | --- | --- | --- |
|  | **n** | **%** | **n** | **%** |  |
|  |  |  |  |  |  |
| **Age (years)** |  |  |  |  |  |
| ≤60 | 112 | 21% | 1053 | 23% | 0.749 |
| >60 to ≤70 | 158 | 30% | 1267 | 28% |  |
| >70 to ≤80 | 198 | 37% | 1601 | 35% |  |
| >80 | 62 | 12% | 672 | 15% |  |
| median | 70 |  | 70 |  |  |
| total | 530 | 100% | 4593 | 100% |  |
|  |  |  |  |  |  |
| **Gender** |  |  |  |  |  |
| male | 295 | 56% | 2854 | 62% | 0.004 |
| female | 235 | 44% | 1739 | 38% |  |
| total | 530 | 100% | 4593 | 100% |  |
|  |  |  |  |  |  |
| **FAB** |  |  |  |  |  |
| RA | 256 | 50% | 1707 | 37% | <0.001 |
| RARS | 57 | 11% | 839 | 18% |  |
| RAEB | 148 | 29% | 1217 | 26% |  |
| RAEB-T | 16 | 3% | 328 | 7% |  |
| CMML | 22 | 4% | 435 | 10% |  |
| Unclassified | 17 | 3% | 67 | 1% |  |
| total | 516 | 97% | 4593 | 100% |  |
|  |  |  |  |  |  |
| **WHO** |  |  |  |  | <0.001 |
| RCUD | 98 | 20% | 639 | 17% |  |
| RARS | 33 | 7% | 507 | 13% |  |
| RCMD | 173 | 36% | 1097 | 29% |  |
| RAEB-1 | 90 | 19% | 627 | 16% |  |
| RAEB-2 | 62 | 13% | 748 | 19% |  |
| MDS (del5q) | 8 | 2% | 143 | 4% |  |
| MDS-U | 17 | 3% | 90 | 2% |  |
| total | 481 | 91% | 3851 | 84% |  |
|  |  |  |  |  |  |
| **IPSS-R** |  |  |  |  | <0.001 |
| Very low | 75 | 14% | 893 | 19% |  |
| Low | 153 | 29% | 1644 | 36% |  |
| Intermediate | 101 | 19% | 882 | 19% |  |
| High | 97 | 18% | 628 | 14% |  |
| Very high | 104 | 20% | 546 | 12% |  |
| total | 530 | 100% | 4593 | 100% |  |
|  |  |  |  |  |  |
| **WPSS-R** |  |  |  |  | <0.001 |
| Very low | 63 | 14% | 822 | 22% |  |
| Low | 100 | 22% | 1036 | 28% |  |
| Intermediate | 112 | 24% | 654 | 17% |  |
| High | 137 | 29% | 916 | 24% |  |
| Very high | 52 | 11% | 325 | 9% |  |
| total | 464 | 88% | 3753 | 82% |  |
|  |  |  |  |  |  |
|  |  |  |  |  |  |
|  |  |  |  |  |  |
|  |  |  |  |  |  |
| **Cytogenetic risk categories (IPSS-R)- cipssr** |  |  |  |  | <0.001 |
| Very good | 15 | 3% | 150 | 3% |  |
| Good | 262 | 50% | 3261 | 71% |  |
| Intermediate | 81 | 15% | 622 | 14% |  |
| poor | 64 | 12% | 197 | 4% |  |
| Very poor | 108 | 20% | 363 | 8% |  |
| total | 530 | 100% | 4593 | 100% |  |
|  |  |  |  |  |  |
| **Number of cytogenetic aberrations** |  |  |  |  | <0.001 |
| 0 | 221 | 42% | 2753 | 73% |  |
| 1 | 114 | 22% | 706 | 19% |  |
| 2 | 57 | 11% | 136 | 4% |  |
| 3 | 29 | 5% | 55 | 1% |  |
| 4 | 17 | 3% | 28 | 1% |  |
| ≥5 | 92 | 17% | 76 | 2% |  |
| total | 530 | 100% | 3754 | 82% |  |
|  |  |  |  |  |  |

**Supplementary Table 2: Patient characteristics** of therapy-related (t-MDS) and primary (p-MDS) myelodysplastic syndromes, untreated patients only
