# Supplementary material for: Therapy-related myelodysplastic syndromes deserve specific diagnostic sub-classification and risk-stratification—an approach to classification of patients with t-MDS
Source: Leukemia. 2020 Jun 29;35(3):835–49. doi: 10.1038/s41375-020-0917-7 (PMC7932916; doi:10.1038/s41375-020-0917-7)
Supplement: Supplementary file 3 — Supplementary Table 3 [file 41375_2020_917_MOESM3_ESM.docx]

| **Score** | **Transformation free survival** | | | | **Overall survival** | | | | **Time to AML** | | | |
| --- | --- | --- | --- | --- | --- | --- | --- | --- | --- | --- | --- | --- |
|  | **t-**  **MDS** | | | **p-MDS** | **t-**  **MDS** | | | **p-MDS** | **t-**  **MDS** | | | **p-MDS** |
|  | **all**  **n=**  **1245** | **treated**  **n=**  **715** | **untreated**  **n=**  **422** | **all**  **n=**  **4593** | **all**  **n=**  **1245** | **treated**  **n=**  **715** | **untreated**  **n=**  **422** | **all**  **n=**  **4593** | **all**  **n=**  **1245** | **treated**  **n=**  **715** | **untreated**  **n=**  **422** | **all**  **n=**  **4593** |
| **FAB** | 0.19 | 0.14 | 0.15 | 0.30 | 0.17 | 0.11 | 0.16 | 0.28 | 0.24 | 0.17 | 0.09 | 0.42 |
| **WHO** | 0.24 | 0.23 | 0.19 | 0.29 | 0.19 | 0.14 | 0.18 | 0.26 | 0.41 | 0.39 | 0.44 | 0.44 |
| **IPSS-R** | 0.37 | 0.32 | 0.46 | 0.41 | 0.38 | 0.33 | 0.45 | 0.40 | 0.36 | 0.28 | 0.48 | 0.53 |
| **WPSS-R** | 0.35 | 0.32 | 0.39 | 0.38 | 0.33 | 0.29 | 0.39 | 0.36 | 0.40 | 0.35 | 0.61 | 0.51 |
| **c-IPSS-R** | 0.30 | 0.27 | 0.32 | 0.23 | 0.32 | 0.32 | 0.30 | 0.23 | 0.23 | 0.16 | 0.54 | 0.28 |
| **Number of aberrations** | 0.29 | 0.26 | 0.32 | 0.13 | 0.32 | 0.33 | 0.30 | 0.13 | 0.22 | 0.14 | 0.52 | 0.14 |
| **Primary diagnosis** | 0.05 |  |  | / | 0.05 |  |  | / | 0.03 |  |  | / |

**Supplementary Table 3: Dxys for the different scoring systems and outcomes presented for t- and p-MDS** **and in addition comparison of the Dxy in treated vs. untreated t-MDS patients** (therapy-related and primary myelodysplastic syndromes): FAB (French-American-British classification), WHO (World Health Organization classification), IPSS-R (International Prognostic Scoring System-revised), WPSS-R (WHO-based Prognostic Scoring System-revised, cipssr (cytogenetic component of the IPSS-R), number of aberrations, and primary diagnosis.
