# Supplementary material for: Therapy-related myelodysplastic syndromes deserve specific diagnostic sub-classification and risk-stratification—an approach to classification of patients with t-MDS
Source: Leukemia. 2020 Jun 29;35(3):835–49. doi: 10.1038/s41375-020-0917-7 (PMC7932916; doi:10.1038/s41375-020-0917-7)
Supplement: Supplementary file 4 — Supplementary Figure Legends [file 41375_2020_917_MOESM4_ESM.docx]

Supplementary Figure Legends:

**Supplementary Figure 1a) Cumulative incidence of death with and without transformation in**

**patients with t-MDS** according to WHO-classification

**1b) Cumulative incidence of death with and without transformation in**

**patients with t-MDS** according to IPSS-R

**1c) Cumulative incidence of death with and without transformation in**

**patients with t-MDS** according to cytogenetic IPSS-R categories

**Supplementary Figure 2a) Outcome of patients with t-MDS** according to FAB-classification

**2b) Outcome of patients with t-MDS** according to WPSS-R

**2c) Outcome of patients with t-MDS** according to IPSS-R plus age (IPSS-R(A))

**2d) Outcome of patients with t-MDS** according to primary diagnosis

**Supplementary Figure 3a) Cumulative incidence of death with and without transformation in**

**patients with t-MDS** **and disease modifying treatment** according to

WHO- classification

**3b) Cumulative incidence of death with and without transformation in**

**patients with t-MDS without disease modifying treatment** according to

WHO-classification

**3c) Cumulative incidence of death with and without transformation in**

**patients with p-MDS** according to WHO-classification

**Supplementary Figure 4a) Cumulative incidence of death with and without transformation in**

**patients with t-MDS** **primary diagnosis hematological disease** according

to WHO-classification

**4b) Cumulative incidence of death with and without transformation in**

**patients with t-MDS primary disease solid tumor** according to WHO-

classification

**4c) Cumulative incidence of death with and without transformation in**

**patients with p-MDS** according to WHO-classification

**Supplementary Figure 5a) Cumulative incidence of death with and without transformation in**

**patients with t-MDS** **vs. p-MDS** according to WHO-classification

**5b) Cumulative incidence of death with and without transformation in**

**patients with t-MDS** **vs. p-MDS** according to IPSS-R

**5c) Cumulative incidence of death with and without transformation in**

**patients with t-MDS vs. p-MDS** according to cytogenetic IPSS-R categories
