# Supplementary figures and images for: Therapy-related myelodysplastic syndromes deserve specific diagnostic sub-classification and risk-stratification—an approach to classification of patients with t-MDS
Source: Leukemia. 2020 Jun 29;35(3):835–49. doi: 10.1038/s41375-020-0917-7 (PMC7932916; doi:10.1038/s41375-020-0917-7)

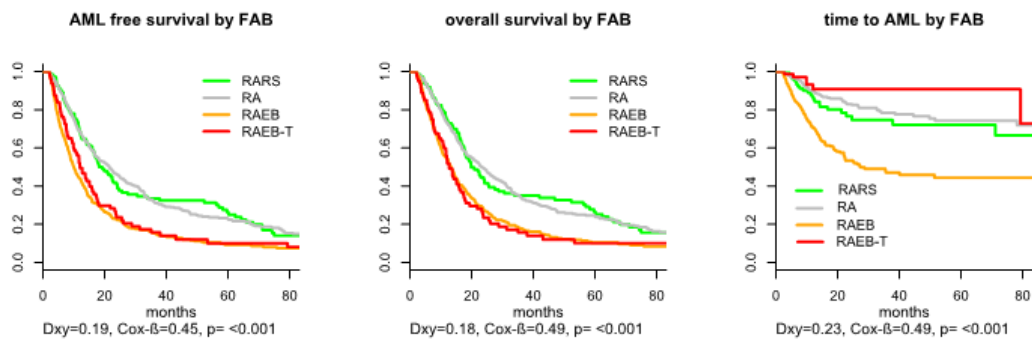

**Suppl Fig 1a)**

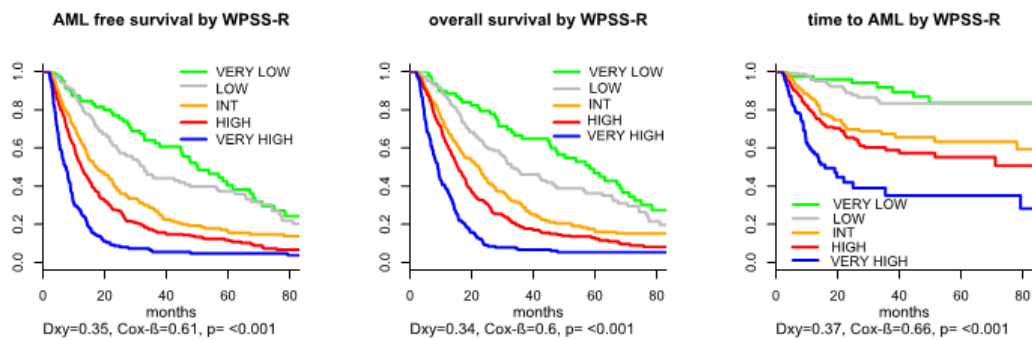

**Suppl Fig 1b)**

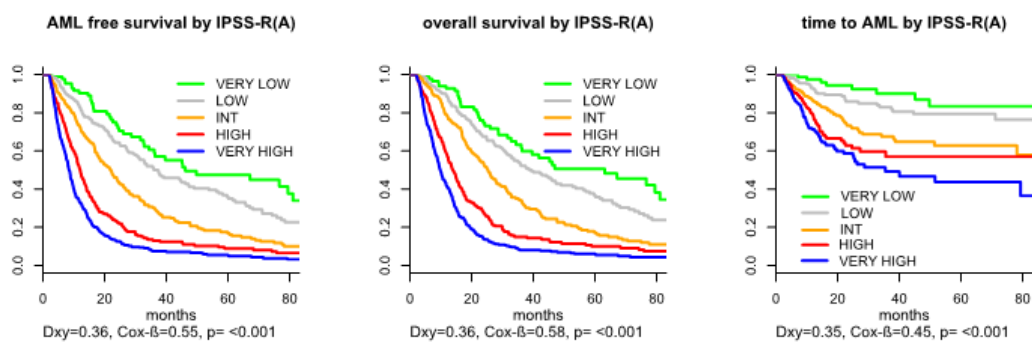

**Suppl Fig 1c)**

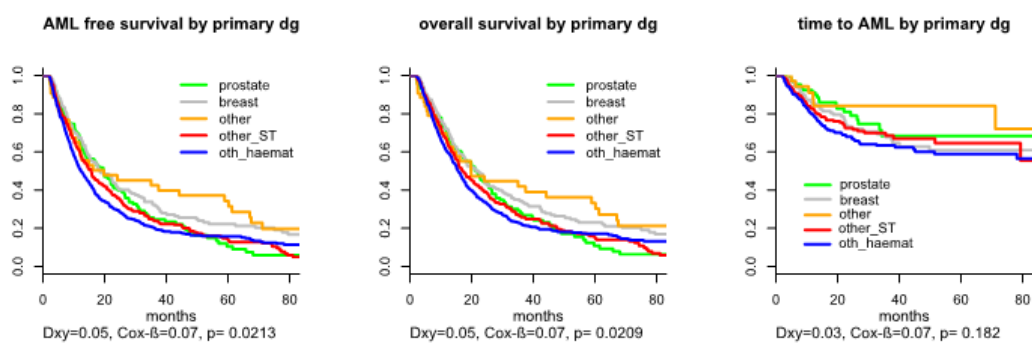

**Suppl Fig 1d)**

Supplement: Supplementary file 5 — Supplementary Figure 1 [file 41375_2020_917_MOESM5_ESM.pdf]

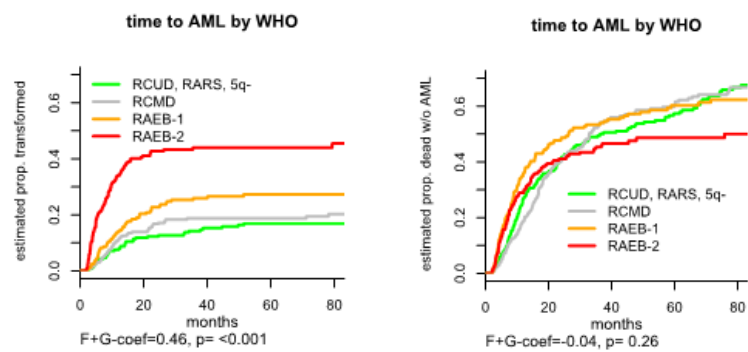

**Suppl Fig 2a)**

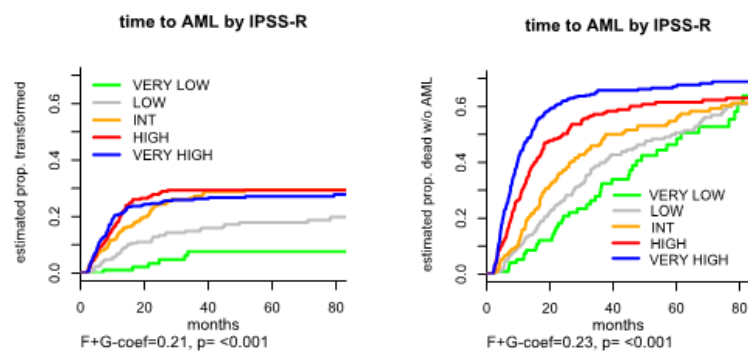

**Suppl Fig 2b)**

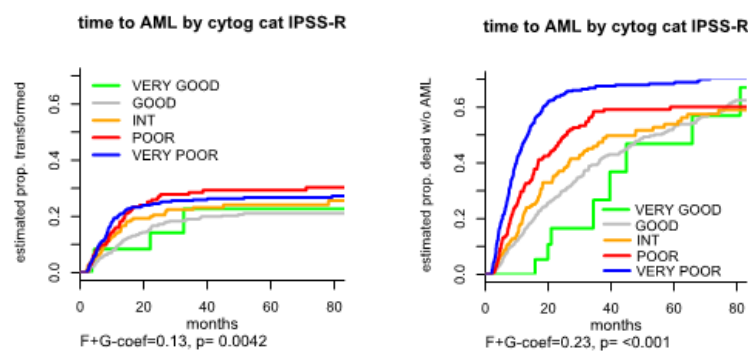

**Suppl Fig 2c)**

Supplement: Supplementary file 6 — Supplementary Figure 2 [file 41375_2020_917_MOESM6_ESM.pdf]

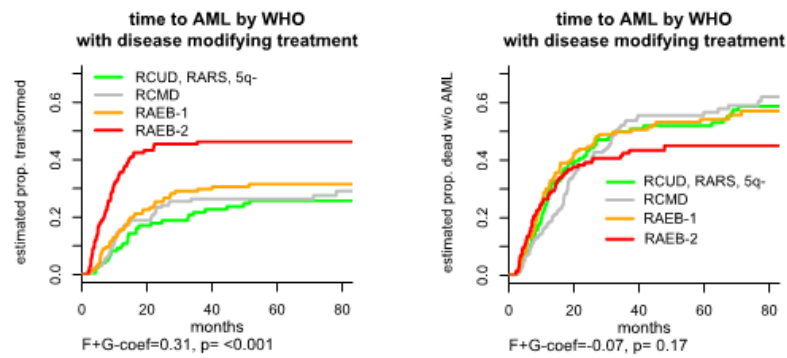

**Suppl Fig 3a)**

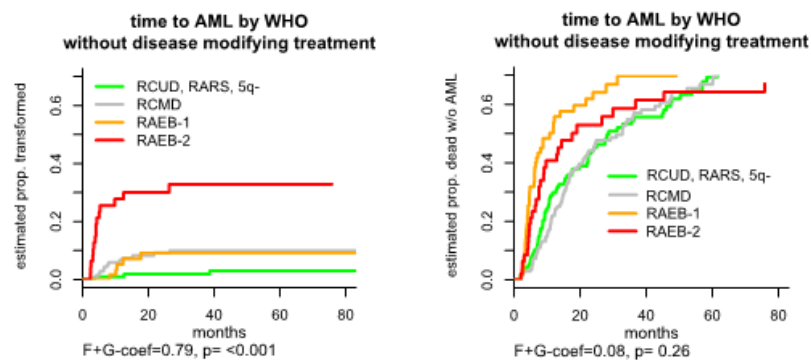

**Suppl Fig 3b)**

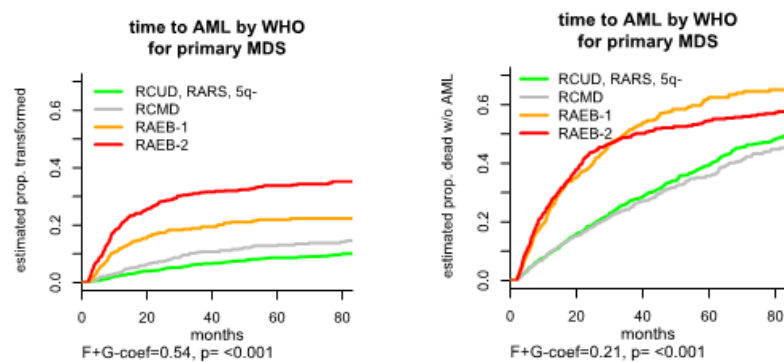

**Suppl Fig 3c)**

Supplement: Supplementary file 7 — Supplementary Figure 3 [file 41375_2020_917_MOESM7_ESM.pdf]

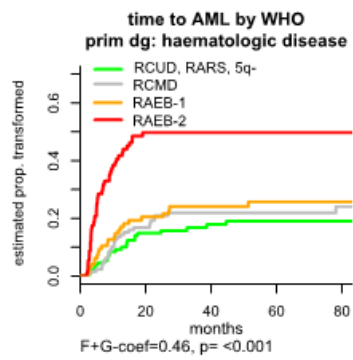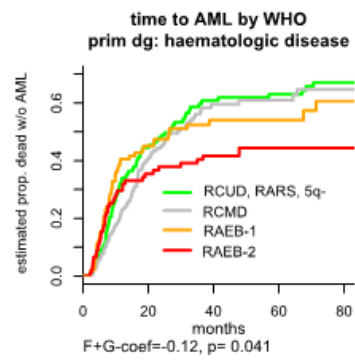

**Suppl Fig 4a)**

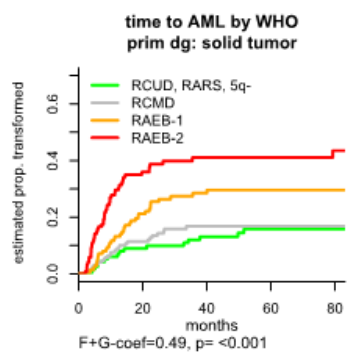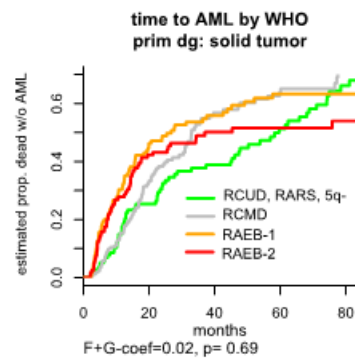

**Suppl Fig 4b)**

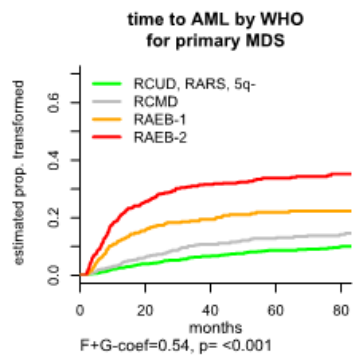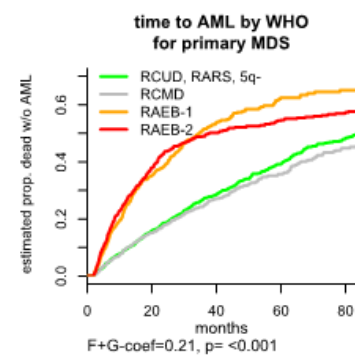

**Suppl Fig 4c)**

Supplement: Supplementary file 8 — Supplementary Figure 4 [file 41375_2020_917_MOESM8_ESM.pdf]

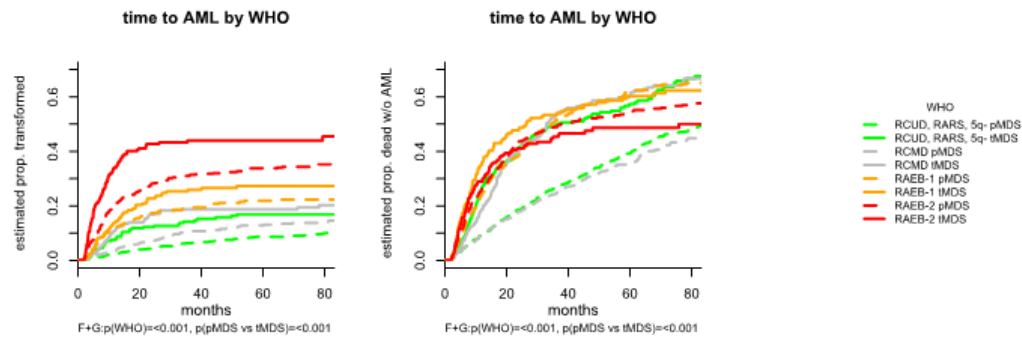

**Suppl Fig 5a)**

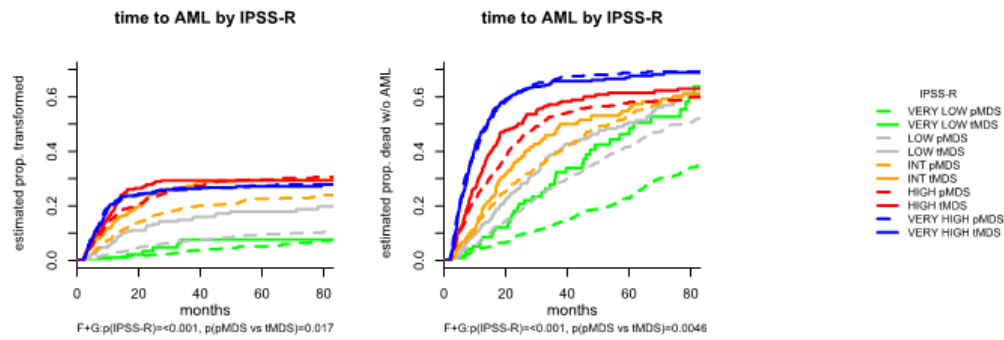

**Suppl Fig 5b)**

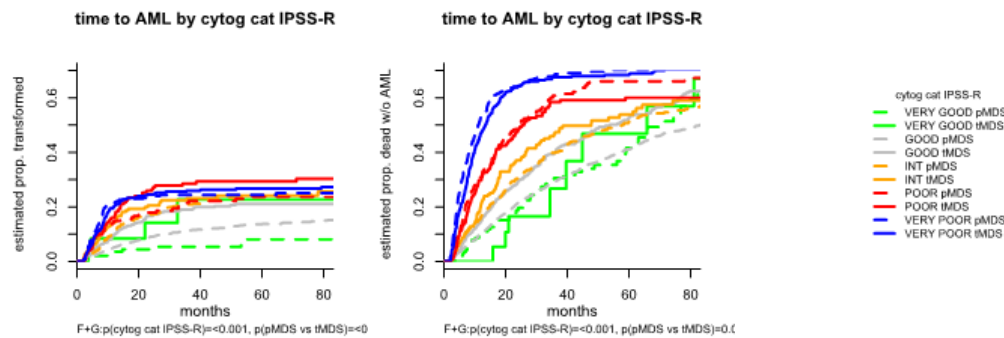

**Suppl Fig 5c)**

Supplement: Supplementary file 9 — Supplementary Figure 5 [file 41375_2020_917_MOESM9_ESM.pdf]
